# Association of Changes in Smoking Intensity With Risk of Dementia in Korea

**DOI:** 10.1001/jamanetworkopen.2022.51506

**Published:** 2023-01-19

**Authors:** Su-Min Jeong, Junhee Park, Kyungdo Han, Juhwan Yoo, Jung Eun Yoo, Cheol Min Lee, Wonyoung Jung, Jinkook Lee, Sang Yun Kim, Dong Wook Shin

**Affiliations:** 1 Department of Medicine, Seoul National University College of Medicine, Seoul, Republic of Korea; 2 Department of Family Medicine, Seoul National University Health Service Center, Seoul, Republic of Korea; 3 Department of Family Medicine, Seoul National University Hospital, Seoul, Republic of Korea; 4 Department of Family Medicine/Supportive Care Center, Samsung Medical Center, Sungkyunkwan University School of Medicine, Seoul, Republic of Korea; 5 Department of Statistics and Actuarial Science, Soongsil University, Seoul, Republic of Korea; 6 Department of Medical Statistics, Catholic University of Korea, Seoul, Republic of Korea; 7 Department of Family Medicine, Healthcare System Gangnam Center, Seoul National University Hospital, Seoul, Republic of Korea; 8 Department of Economics, Center for Economic and Social Research, University of Southern California, Los Angeles; 9 RAND Corporation, Santa Monica, California; 10 Department of Neurology, Seoul National University Bundang Hospital, Seongnam, Republic of Korea; 11 Seoul National University College of Medicine, Seongnam, Republic of Korea; 12 Department of Clinical Research Design and Evaluation, Samsung Advanced Institute for Health Science and Technology, Sungkyunkwan University, Seoul, Republic of Korea

## Abstract

**Importance:**

Several observational studies have reported that smoking cessation is associated with a lower risk of dementia. However, no studies have examined the association between change in smoking intensity and risk of dementia.

**Objective:**

To investigate the association between a change in smoking intensity, including smoking reduction and smoking cessation, and risk of all dementia.

**Design, Setting, and Participants:**

This cohort study used data from the National Health Insurance Service database of Korea. The cohort included participants 40 years or older who underwent biennial health examinations (2009 and 2011) and had current smoking status at the first health examination. The cohort was followed up until December 31, 2018, and statistical analysis was performed between July and December 2021.

**Exposures:**

Change in smoking intensity from baseline was defined operationally as follows: quitters (stopped smoking), reducers I (decreased number of cigarettes smoked per day by ≥50%), reducers II (decreased number of cigarettes smoked per day by 20%-50%), sustainers (maintained [decreased or increased] number of cigarettes smoked per day by less than 20%), or increasers (increased number of cigarettes smoked per day by ≥20%).

**Main Outcomes and Measures:**

The primary outcome was newly diagnosed dementia, which was identified by prescribed antidementia medications with concomitant *International Statistical Classification of Diseases and Related Health Problems, Tenth Revision* diagnosis codes for dementia. Adjusted hazard ratios and 95% CIs were used to determine the association between change in smoking intensity and incidence of dementia, including Alzheimer disease (AD) and vascular dementia (VaD).

**Results:**

A total of 789 532 participants (756 469 males [95.8%]; mean [SD] age, 52.2 [8.5] years) were included. During a median (IQR) follow-up period of 6.3 (6.1-6.6) years, 11 912 dementia events, including 8800 AD and 1889 VaD events, were identified. Overall, participants in the quitter group had a significantly lower risk of all dementia (adjusted hazard ratio [aHR], 0.92; 95% CI, 0.87-0.97) compared with those in the sustainer group. Those in the reducer I (aHR, 1.25; 95% CI, 1.18-1.33) and increaser (aHR, 1.12; 95% CI, 1.06-1.18) groups had a significantly higher risk of all dementia compared with those in the sustainer group.The patterns for AD and VaD remained consistent with patterns for all dementia.

**Conclusions and Relevance:**

The results of this study showed that smoking cessation was associated with a lower risk of dementia compared with sustained smoking intensity, while smoking reduction was associated with a higher risk. Smoking cessation should be emphasized in efforts to reduce the disease burden of dementia.

## Key Points

**Question:**

Are changes in smoking intensity, such as cessation or reduction, associated with a lower the risk of dementia?

**Findings:**

In this cohort study of 789 532 participants in Korea, smoking cessation was associated with decreased risk of dementia compared with sustained smoking. In addition, an increased risk of dementia was found in patients who reduced or increased cigarettes smoked per day compared with those who sustained a consistent rate of consumption.

**Meaning:**

Findings of this study suggest that smoking cessation instead of smoking reduction should be emphasized in reducing the disease burden of dementia.

## Teaser

This cohort study evaluates the incidence of dementia, including Alzheimer disease and vascular dementia, among a mostly male population of current and previous cigarette smokers in Korea.

## Introduction

With an aging population worldwide, approximately 152 million individuals are expected to be affected by dementia by 2050.^1^ Smoking is a well-known risk factor for dementia and accounts for the third highest population-attributable percentage of dementia cases (5.2%), following low educational level and hearing loss.^2^

Several observational studies have reported that smoking cessation is associated with a decreased risk of dementia^3,4,5,6,7^(eTable 1 in Supplement 1). Several studies based on baseline smoking status showed that past smokers had a lower risk of dementia than current smokers.^4,6^ However, these studies sampled a relatively small proportion of the population (<2000 dementia cases),^3,4,5,6,7^ were limited to specific populations (eg, men^3^ or older individuals with a mean age of 72 years),^6^ or did not assess or adjust for intensity of smoking.^3,4,5,6,7^

In addition, no previous study, to our knowledge, has examined the association between smoking reduction and risk of dementia.^8^ Considering the evidence of a dose-response association between smoking intensity and health outcomes,^9^ smoking reduction may be associated with a decreased risk of dementia. However, most previous studies did not find a significant association between smoking reduction and various health outcomes^10,11,12,13,14^ Therefore, it is unclear whether smoking reduction is associated with a decreased risk of dementia. Thus, the goal of the current study was to investigate the association between change in smoking intensity, including smoking reduction and smoking cessation, and risk of all dementia.

## Methods

### Design, Setting, and Population

This population-based, retrospective cohort study used data from the National Health Insurance Service (NHIS), a mandatory universal insurance system that covers the entire population in Korea. This study was approved by the Samsung Medical Center Institutional Review Board, which waived the informed consent requirement because the study used retrospective anonymized data. We followed the Strengthening the Reporting of Observational Studies in Epidemiology (STROBE) reporting guideline.

The NHIS database contains demographic data and links the data to health care claims. The NHIS database also contains data from the national health checkup program, which covers all employed individuals and beneficiaries 40 years or older, including data from self-administered health questionnaires, anthropometric measurements, and laboratory tests.^15^

We identified from the NHIS database participants 40 years or older who underwent biennial health examinations in 2009 and 2011. We selected participants with current smoking status (n = 1 006 855) at the first health examination, according to the definition of the World Health Organization.^16^ Participants who were diagnosed with any cancer (n = 15 629), stroke (n = 42 165), myocardial infarction (n = 19 544), or dementia (n = 986) prior to the second health examination period (2011) were excluded. We applied a 1-year lag time to reduce the implications of reverse causality by excluding participants who were diagnosed with cancer (n = 9328), stroke (n = 6426), myocardial infarction (n = 2958), or dementia (n = 714) or participants who died (n = 1834) within 1 year after the second health examination (2011). Those who had missing information on variables (eTable 2 in Supplement 1) used in this study were excluded, resulting in the inclusion of 789 532 individuals in the study cohort.

### Definition of Change in Cigarette Smoking Intensity

Information on smoking status and change in smoking intensity was obtained from self-administered questionnaires during the health checkup. Participants who acknowledged having smoked at least 100 cigarettes in their lifetime, based on the World Health Organization definition,^16^ were asked about current smoking status. Then, current smokers were questioned on the duration of smoking and mean number of cigarettes smoked per day (eTable 3 in Supplement 1). According to cigarette smoking intensity at the time of the first health examination (2009), participants were categorized as mild smokers (<10 cigarettes per day), moderate smokers (10-19 cigarettes per day), and heavy smokers (≥20 cigarettes per day).^17^

In this study, change in cigarette smoking intensity was identified based on (1) relative change in the number of cigarettes smoked per day (reducer or increaser group) and (2) categorical change in the level of smoking intensity (eg, mild to heavy smoking status). Participants were categorized into 5 groups based on a relative change in smoking intensity between the first (2009) and the second (2011) health examinations: quitter, reducer I, reducer II, sustainer, or increaser, based on definitions used in previous studies.^18,19^ Quitters were defined as those who completely stopped smoking (ie, current smokers in 2009 who became former smokers in 2011). The reducer group was divided into subcategories to evaluate any association according to the degree of smoking reduction: reducers I were defined as those who decreased the number of cigarettes per day by 50% or more, while reducers II were those who decreased the number of cigarettes per day by 20% to 50%. Sustainers were defined as those who maintained (increased or decreased) the number of cigarettes per day by less than 20%. Increasers were defined as those who increased the number of cigarettes per day by 20% or more.

### Outcomes and Follow-up

The primary end point was newly diagnosed dementia, identified on the basis of prescribed antidementia medications (rivastigmine, galantamine, memantine, or donepezil) along with *International Statistical Classification of Diseases and Related Health Problems, Tenth Revision (ICD-10)* codes for dementia (F00, F01, F02, F03, G30, or G31).^20,21,22,23,24^ For valid medical expense claims submitted to the NHIS, physicians need to document the evidence for cognitive dysfunction according to strict criteria. In particular, a Mini-Mental State Examination score of 26 or lower (scale: 0-30, with the lowest score indicating dementia) and either a Clinical Dementia Rating of 1 or higher (scale: 0-5, with the highest rating indicating dementia) or Global Deterioration Scale score of 3 or higher (scale: 1-7, with the highest score indicating dementia) were required.^25^ Patients were categorized as having Alzheimer disease (AD; *ICD-10* code F00 or G30), vascular dementia (VaD; *ICD-10* code F01), or another type of dementia.^3^ The cohort was followed up after 1 year of lag time from the second health examination (2011) until the end of the study period (December 31, 2018). Information on covariates is provided in the eMethods in Supplement 1.

### Statistical Analysis

Continuous variables were presented as mean (SD), and categorical variables were presented as number (percentage). Hazard ratios (HR) with 95% CIs for all dementia and subtypes (AD or VaD) were calculated using a Cox proportional hazards regression model. The proportional hazards assumption was tested using Schoenfeld residuals. Multivariable models were adjusted for age; sex; household income; alcohol consumption; regular physical activity; area of residence; comorbidities, such as hypertension, diabetes, dyslipidemia, and chronic kidney disease; and body mass index. Analysis was performed using 2 reference groups: (1) sustainers (relative change of <20% in number of cigarettes smoked per day) and (2) those in the same category of smoking intensity (eg, both in the mild smoker category for 2009 and 2011 health examinations) (eTable 4 in Supplement 1). We analyzed the restricted cubic spline curve to assess the association between change in smoking intensity treated as a continuous variable and the incidence of dementia.

In the sensitivity analysis, a subdistribution hazard model regression using the Fine-Gray methods was performed to estimate the subdistribution HR for dementia incidence, accounting for death as a competing event.^26^ Multiple imputation was also used to account for missing covariate data.^27^ Stratification analyses by smoking intensity at the first health examination, with age, sex, and alcohol consumption as confounding factors, were performed to assess the association between change in smoking intensity and incidence of dementia.

Statistical analyses were performed between July and December 2021, using SAS, version 9.4 (SAS Institute Inc). Two-sided *P* < .05 was considered statistically significant.

## Results

### Characteristics of the Study Participants

The cohort comprised 789 532 participants (756 469 males [95.8%], 33 063 females [4.2%]; mean [SD] age, 52.2 [8.5] years). Table 1 shows the baseline characteristics during the 2011 health examination, according to relative changes in smoking intensity (quitters, reducers I, reducers II, sustainers, and increasers). We compared baseline characteristics of individuals who were included vs excluded in the current study (eTable 5 in Supplement 1).

**Table 1.** Baseline Characteristics of the Study Population in 2011

Variable	All participants (n = 789 532)	Participant group^a^					*P* value
		Quitter (n = 114 959)	Reducer I (n = 60 767)	Reducer II (n = 111 890)	Sustainer (n = 376 393)	Increaser (n = 125 523)	
Age, mean (SD), y	52.2 (8.5)	53.3 (8.8)	53.9 (9.4)	51.9 (8.3)	52.0 (8.3)	52.0 (8.6)	<.001
Sex, No. (%)							
Female	33 063 (4.2)	4811 (4.2)	4072 (6.7)	4501 (4.0)	12 765 (3.4)	6914 (5.5)	<.001
Male	756 469 (95.8)	110 148 (95.8)	56 695 (93.3)	107 389 (96.0)	363 628 (96.6)	118 609 (94.5)	<.001
Alcohol consumption, No. (%)							
None	203 562 (25.8)	32 916 (28.6)	17 800 (29.3)	28 038 (25.1)	93 169 (24.8)	31 639 (25.2)	<.001
Mild	278 957 (35.3)	43 483 (37.8)	23 939 (39.4)	41 800 (37.4)	128 661 (34.2)	41 074 (32.7)	NA
Moderate	178 391 (22.6)	22 745 (19.8)	11 900 (19.6)	25 469 (22.8)	89 670 (23.8)	28 607 (22.8)	NA
Heavy	128 622 (16.3)	15 815 (13.8)	7128 (11.7)	16 583 (14.8)	64 893 (17.2)	24 203 (19.3)	NA
Regular physical activity, No. (%)							
None	632 538 (80.1)	85 519 (74.4)	47 665 (78.4)	89 733 (80.2)	307 234 (81.6)	102 387 (81.6)	<.001
Regular	156 994 (19.9)	29 440 (25.6)	13 102 (21.6)	22 157 (19.8)	69 159 (18.4)	23 136 (18.4)	<.001
Anthropometrics, mean (SD)							
Height, cm	168.4 (6.4)	168.4 (6.4)	167.5 (6.8)	168.5 (6.4)	168.5 (6.3)	168.1 (6.6)	<.001
Weight, kg	68.0 (10.2)	69.5 (10.0)	67.1 (10.5)	67.9 (10.2)	67.8 (10.1)	67.6 (10.4)	<.001
Waist circumference, cm	83.5 (7.7)	84.8 (7.5)	83.4 (7.9)	83.4 (7.7)	83.3 (7.7)	83.3 (7.8)	<.001
BMI	23.9 (3.0)	24.5 (2.9)	23.8 (3.0)	23.9 (3.0)	23.8 (3.0)	23.9 (3.0)	<.001
Blood pressure, mmHg							
Systolic	124.4 (14.3)	125.4 (14.2)	124.8 (14.7)	124.3 (14.2)	124.3 (14.3)	124.2 (14.4)	<.001
Diastolic	78.2 (9.8)	78.7 (9.8)	78.1 (9.9)	78.2 (9.8)	78.1 (9.8)	78.0 (9.9	<.001
Comorbidities, No. (%)							
Hypertension	241 806 (30.6)	39 122 (34.0)	19 900 (32.8)	33 599 (30.0)	111 891 (29.7)	37 294 (29.7)	<.001
Diabetes	103 139 (13.1)	15 738 (13.7)	8694 (14.3)	14 194 (12.7)	48 059 (12.8)	16 454 (13.1)	<.001
Dyslipidemia	160 062 (20.3)	27 356 (23.8)	12 504 (20.6)	22 306 (19.9)	73 550 (19.5)	24 346 (19.4)	<.001
CKD	31 577 (4.0)	5707 (5.0)	2660 (4.4)	4401 (3.9)	14 118 (3.8)	4691 (3.7)	<.001
Laboratory findings							
Glucose, mg/dL	102.2 (27.6)	103.1 (27.3)	102.7 (28.6)	101.9 (27.3)	101.9 (27.4)	102.3 (28.8)	<.001
Total cholesterol, mg/dL	198.2 (36.1)	200.4 (36.9)	196.7 (36.6)	197.8 (36.0)	197.9 (35.9)	197.4 (36.0)	<.001
HDL, mg/dL	52.1 (16.8)	52.4 (16.5)	52.2 (17.2)	51.8 (16.5)	51.9 (17.0)	52.3 (16.7)	<.001
LDL, mg/dL	114.5 (35.3)	116.7 (34.9)	113.1 (35.5)	114.2 (35.3)	114.2 (35.4)	113.6 (35.2	<.001
GFR, mL/min/1.73m^2^	88.9 (37.4)	87.1 (38.4)	89.1 (39.1)	88.8 (34.5)	89.2 (37.9)	89.6 (37.0)	<.001
Urban residency, No. (%)	355 838 (45.1)	52 520 (45.7)	26 382 (43.4)	51 107 (45.7)	169 748 (45.1)	56 081 (44.7)	<.001
Household income, No. (%)							
Quartile 1 (lowest)	147 697 (18.7)	20 380 (17.7)	12 853 (21.2)	21 159 (18.9)	69 275 (18.4)	24 030 (19.1)	<.001
Quartile 2	134 155 (17.0)	17 867 (15.5)	11 502 (18.9)	18 844 (16.8)	63 258 (16.8)	22 684 (18.1)	NA
Quartile 3	213 459 (27.0)	29 313 (25.5)	16 158 (26.6)	30 149 (27.0)	103 650 (27.5)	34 189 (27.2)	NA
Quartile 4 (highest)	294 221 (37.3)	47 399 (41.2)	20 254 (33.3)	41 738 (37.3)	140 210 (37.3)	44 620 (35.6)	NA
Smoking status in 2009, No. (%)							
Mild: <10 cigarettes/d	69 292 (8.8)	18 417 (16.0)	2694 (4.4)	6280 (5.6)	14 693 (3.9)	27 208 (21.7)	<.001
Moderate: 10-19 cigarettes/d	295 770 (37.5)	47 357 (41.2)	13 062 (21.5)	38 056 (34.0)	125 764 (33.4)	71 531 (57.0)	NA
Heavy: ≥20 cigarettes/d	424 470 (53.8)	49 185 (42.8)	45 011 (74.1)	67 554 (60.4)	235 936 (62.7)	26 784 (21.3)	NA
Duration of smoking in 2009, y, No. (%)							
<5	18 674 (2.4)	5541 (4.8)	1474 (2.4)	1776 (1.6)	5689 (1.5)	4194 (3.3)	<.001
5-9	18 229 (2.3)	3974 (3.5)	1616 (2.7)	2054 (1.8)	6570 (1.8)	4015 (3.2)	NA
10-19	119 613 (15.2)	19 550 (17.0)	9391 (15.5)	15 664 (14.0)	51 711 (13.7)	23 297 (18.6)	NA
20-29	361 970 (45.9)	46 801 (40.7)	24 605 (40.5)	53 676 (48.0)	181 015 (48.1)	55 873 (44.5)	NA
≥30	271 046 (34.3)	39 093 (34.0)	23 681 (39.0)	38 720 (34.6)	131 408 (34.9)	38 144 (30.4)	NA
Pack-years of smoking in 2009, No. (%)							
<10	129 430 (16.4)	29 734 (25.9)	7473 (12.3)	12 045 (10.8)	41 525 (11.0)	38 653 (30.8)	<.001
10 to <20	227 456 (28.8)	34 095 (29.7)	12 407 (20.4)	28 597 (25.6)	102 102 (27.1)	50 255 (40.0)	NA
20 to <30	222 228 (28.2)	26 063 (22.7)	17 264 (28.4)	30 099 (26.9)	126 763 (33.7)	22 039 (17.6)	NA
≥30	210 418 (26.7)	25 067 (21.8)	23 623 (38.9)	41 149 (36.8)	106 003 (28.2)	14 576 (11.6)	NA
Smoking status in 2011, No. (%)							
None: 0 cigarettes/d	114 959 (14.6)	114 959 (100)	NA	NA	NA	NA	<.001
Mild: <10 cigarettes/d	60 417 (7.7)	NA	22 110 (36.4)	15 576 (13.9)	14 750 (3.9)	7981 (6.4)	NA
Moderate: 10-19 cigarettes/d	262 132 (33.2)	NA	33 173 (54.6)	65 051 (58.1)	125 992 (33.5)	37 916 (30.2)	NA
Heavy: ≥20 cigarettes/d	352 024 (44.6)	NA	5484 (9.0)	31 263 (27.9)	235 651 (62.6)	79 626 (63.4)	NA
Duration of smoking in 2011, y, No. (%)							
<5	125 034 (15.8)	114 959 (100)	2424 (4.0)	1564 (1.4)	3838 (1.0)	2249 (1.8)	<.001
5-9	13 652 (1.7)	0	2387 (3.9)	2293 (2.0)	5907 (1.6)	3065 (2.4)	NA
10-19	83 989 (10.6)	0	10 851 (17.9)	14 961 (13.4)	41 940 (11.1)	16 237 (12.9)	NA
20-29	294 061 (37.2)	0	21 620 (35.6)	49 849 (44.6)	168 298 (44.7)	54 294 (43.3)	NA
≥30	272 796 (34.6)	0	23 485 (38.7)	43 223 (38.6)	156 410 (41.6)	49 678 (39.6)	NA
Pack-years of smoking in 2011, No. (%)							
<10	124 411 (15.8)	25 093 (21.8)	27 060 (44.5)	20 627 (18.4)	36 489 (9.7)	15 142 (12.1)	<.001
10 to <20	224 677 (28.5)	31 292 (27.2)	22 920 (37.7)	45 227 (40.4)	96 270 (25.6)	28 968 (23.1)	NA
20 to <30	214 052 (27.1)	26 271 (22.9)	7405 (12.2)	25 659 (22.9)	121 748 (32.4)	32 969 (26.3)	NA
≥30	226 392 (28.7)	32 303 (28.1)	3382 (5.6)	20 377 (18.2)	121 886 (32.4)	48 444 (38.6)	NA

Abbreviations: BMI, body mass index (calculated as weight in kilograms divided by height in meters squared); CKD, chronic kidney disease; GFR, glomerular filtration rate; HDL, high-density lipoprotein; LDL, low-density lipoprotein; NA, not applicable.

SI conversion factor: To convert glucose to millimoles per liter, multiply by 0.0555; HDL, LDL, and total cholesterol to millimoles per liter, multiply by 0.0259.

^a^ Quitters were defined as those who stopped smoking; reducers, those who decreased the number of cigarettes smoked per day by 50% or more (reducers I) or by 20% to 50% (reducers II); sustainers, those who maintained (decreased or increased) the number of cigarettes smoked per day by less than 20%; and increasers, those who increased the number of cigarettes smoked per day by 20% or more.

At the 2009 health examination, 53.8% of participants were heavy smokers, 37.5% were moderate smokers, and 8.8% were mild smokers. By the 2011 health examination, 14.6% of participants had quit smoking and 21.9% had reduced their smoking (7.7% in the reducer I group and 14.2% in the reducer II group). However, 15.9% of participants increased the number of cigarettes they smoked during the 2-year interval. Most of the participants (80.2%) had smoked for 20 years or more: 45.9% for 20 to 29 years and 34.3% for 30 years or more (Table 2). Those in the quitter and increaser groups were more likely to be mild to moderate smokers, while most in the reducer group were heavy smokers.

**Table 2.** Association Between Relative Changes in Cigarette Smoking Intensity and Risk of Dementia

Smoking status in 2009^a^	Smoking status in 2011^a^	Participants, No. (%)	Dementia cases, No.	Duration, person-year^b^	IR	Crude model HR (95% CI)	Age-adjusted model aHR (95% CI)^c^	Multivariate model aHR (95% CI)^d^	Multivariate competing risk SHR (95% CI)
**All dementia (total)**									
All current smokers (n = 789 532)	Quitter	114 959 (14.6)	1730	722 407	2.4	1.13 (1.07-1.20)	0.87 (0.83-0.92)	0.92 (0.87-0.97)	0.92 (0.87-0.97)
	Reducer I	60 767 (7.7)	1585	376 954	4.2	1.99 (1.88-2.11)	1.26 (1.19-1.33)	1.25 (1.18-1.33)	1.24 (1.17-1.32)
	Reducer II	111 890 (14.2)	1570	699 937	2.2	1.07 (1.01-1.13)	1.05 (0.99-1.11)	1.06 (1.00-1.12)	1.05 (0.99-1.12)
	Sustainer	376 393 (47.7)	4958	2 355 282	2.1	1 [Reference]	1 [Reference]	1 [Reference]	1 [Reference]
	Increaser	125 523 (15.9)	2069	783 225	2.6	1.26 (1.19-1.32)	1.13 (1.07-1.20)	1.12 (1.06-1.18)	1.11 (1.05-1.17)
Mild smokers: <10 CPD (n = 69 292)	Quitter	18 417 (26.6)	435	115 189	3.8	0.84 (0.73-0.96)	0.97 (0.84-1.11)	1.00 (0.87-1.15)	1.02 (0.89-1.18)
	Reducer I	2694 (3.9)	116	16 587	7.0	1.55 (1.26-1.91)	1.24 (1.01-1.52)	1.24 (1.01-1.52)	1.29 (1.04-1.61)
	Reducer II	6280 (9.1)	282	38 662	7.3	1.62 (1.39-1.89)	1.18 (1.02-1.38)	1.19 (1.02-1.38)	1.19 (1.01-1.40)
	Sustainer	14 693 (21.2)	411	91 292	4.5	1 [Reference]	1 [Reference]	1 [Reference]	1 [Reference]
	Increaser	27 208 (39.3)	838	168 476	5.0	1.11 (0.98-1.24)	1.17 (1.04-1.32)	1.17 (1.04-1.32)	1.20 (1.06-1.36)
Moderate smokers: 10-19 CPD (n = 295 770)	Quitter	47 357 (16.0)	637	298 035	2.1	0.97 (0.89-1.06)	0.85 (0.77-0.93)	0.88 (0.81-0.97)	0.88 (0.80-0.97)
	Reducer I	13 062 (4.4)	424	80 973	5.2	2.38 (2.14-2.64)	1.35 (1.22-1.51)	1.30 (1.17-1.45)	1.28 (1.14-1.44)
	Reducer II	38 056 (12.9)	654	237 973	2.7	1.25 (1.14-1.37)	1.10 (1.01-1.12)	1.12 (1.02-1.22)	1.13 (1.03-1.24)
	Sustainer	125 764 (42.5)	1731	788 212	2.2	1 [Reference]	1 [Reference]	1 [Reference]	1 [Reference]
	Increaser	71 531 (24.2)	970	447 233	2.2	0.99 (0.91-1.07)	1.16 (1.08-1.26)	1.15 (1.06-1.24)	1.14 (1.05-1.23)
Heavy smokers: ≥20 CPD (n = 424 470)	Quitter	49 185 (11.6)	658	309 183	2.1	1.11 (1.02-1.21)	0.86 (0.79-0.94)	0.92 (0.85-1.01)	0.92 (0.84-1.00)
	Reducer I	45 011 (10.6)	1045	279 395	3.7	1.95 (1.82-2.10)	1.21 (1.12-1.30)	1.21 (1.13-1.30)	1.19 (1.11-1.29)
	Reducer II	67 554 (15.9)	634	423 302	1.5	0.79 (0.72-0.86)	0.96 (0.88-1.05)	0.98 (0.90-1.07)	0.96 (0.88-1.05)
	Sustainer	235 936 (55.6)	2816	1 475 778	1.9	1 [Reference]	1 [Reference]	1 [Reference]	1 [Reference]
	Increaser	26 784 (6.3)	261	167 517	1.6	0.82 (0.72-0.93)	1.08 (0.95-1.23)	1.06 (0.93-1.20)	1.05 (0.93-1.20)
**Alzheimer dementia**									
All current smokers (n = 789 532)	Quitter	114 959 (14.6)	1314	722 407	1.8	1.17 (1.10-1.25)	0.89 (0.84-0.95)	0.94 (0.88-1.00)	0.94 (0.88-1.01)
	Reducer I	60 767 (7.7)	1191	376 954	3.2	2.04 (1.88-2.11)	1.25 (1.17-1.33)	1.24 (1.16-1.32)	1.23 (1.14-1.32)
	Reducer II	111 890 (14.2)	1162	699 937	1.7	1.08 (1.01-1.15)	1.05 (0.98-1.12)	1.06 (0.99-1.13)	1.05 (0.98-1.13)
	Sustainer	376 393 (47.7)	3636	2 355 282	1.5	1 [Reference]	1 [Reference]	1 [Reference]	1 [Reference]
	Increaser	125 523 (15.9)	1497	783 225	1.9	1.24 (1.17-1.32)	1.10 (1.03-1.17)	1.08 (1.02-1.15)	1.08 (1.01-1.15)
Mild smokers: <10 CPD (n = 69 292)	Quitter	18 417 (26.6)	357	115 189	3.1	0.89 (0.76-1.03)	1.03 (0.89-1.20)	1.08 (0.92-1.25)	1.13 (0.96-1.32)
	Reducer I	2694 (3.9)	93	16 587	5.6	1.60 (1.27-2.02)	1.26 (1.00-1.59)	1.26 (1.00-1.59)	1.34 (1.05-1.71)
	Reducer II	6280 (9.1)	221	38 662	5.7	1.64 (1.38-1.94)	1.18 (0.99-1.40)	1.18 (0.99-1.40)	1.19 (0.99-1.44)
	Sustainer	14 693 (21.2)	319	91 292	3.5	1 [Reference]	1 [Reference]	1 [Reference]	1 [Reference]
	Increaser	27 208 (39.3)	636	168 476	3.8	1.08 (0.95-1.24)	1.15 (1.01-1.32)	1.15 (1.00-1.31)	1.19 (1.03-1.37)
Moderate smokers: 10-19 CPD (n = 295 770)	Quitter	47 357 (16.0)	469	298 035	1.6	0.96 (0.86-1.07)	0.84 (0.75-0.93)	0.88 (0.79-0.98)	0.89 (0.79-0.99)
	Reducer I	13 062 (4.4)	337	80 973	4.2	2.54 (2.25-2.86)	1.40 (1.24-1.58)	1.33 (1.18-1.51)	1.32 (1.15-1.51)
	Reducer II	38 056 (12.9)	492	237 973	2.1	1.27 (1.14-1.41)	1.11 (1.00-1.23)	1.13 (1.01-1.25)	1.15 (1.03-1.28)
	Sustainer	125 764 (42.5)	1286	788 212	1.6	1 [Reference]	1 [Reference]	1 [Reference]	1 [Reference]
	Increaser	71 531 (24.2)	681	447 233	1.5	0.94 (0.85-1.03)	1.12 (1.02-1.23)	1.10 (1.01-1.21)	1.10 (1.00-1.21)
Heavy smokers: ≥20 CPD (n = 424 470)	Quitter	49 185 (11.6)	488	309 183	1.6	1.14 (1.03-1.26)	0.87 (0.79-0.96)	0.93 (0.84-1.03)	0.92 (0.83-1.02)
	Reducer I	45 011 (10.6)	761	279 395	2.7	1.97 (1.81-2.14)	1.16 (1.07-1.26)	1.17 (1.07-1.27)	1.14 (1.05-1.25)
	Reducer II	67 554 (15.9)	449	423 302	1.1	0.77 (0.70-0.86)	0.96 (0.87-1.07)	0.98 (0.89-1.09)	0.95 (0.85-1.06)
	Sustainer	235 936 (55.6)	2031	1 475 778	1.4	1 [Reference]	1 [Reference]	1 [Reference]	1 [Reference]
	Increaser	26 784 (6.3)	180	167 517	1.1	0.78 (0.67-0.91)	1.07 (0.92-1.24)	1.05 (0.90-1.22)	1.04 (0.89-1.22)
**Vascular dementia**									
All current smokers (n = 789 532)	Quitter	114 959 (14.6)	245	722 407	0.3	0.98 (0.85-1.13)	0.80 (0.70-0.93)	0.84 (0.73-0.97)	0.84 (0.73-0.97)
	Reducer I	60 767 (7.7)	232	376 954	0.6	1.78 (1.54-2.06)	1.28 (1.11-1.48)	1.28 (1.11-1.49)	1.24 (1.07-1.44)
	Reducer II	111 890 (14.2)	256	699 937	0.4	1.06 (0.92-1.22)	1.06 (0.92-1.22)	1.07 (0.93-1.24)	1.07 (0.93-1.24)
	Sustainer	376 393 (47.7)	814	2 355 282	0.3	1 [Reference]	1 [Reference]	1 [Reference]	1 [Reference]
	Increaser	125 523 (15.9)	342	783 225	0.4	1.26 (1.11-1.43)	1.20 (1.06-1.36)	1.19 (1.05-1.35)	1.18 (1.04-1.34)
Mild smokers: <10 CPD (n = 69 292)	Quitter	18 417 (26.6)	44	115 189	0.4	0.59 (0.40-0.87)	0.65 (0.44-0.96)	0.65 (0.44-0.96)	0.65 (0.43-0.97)
	Reducer I	2694 (3.9)	11	16 587	0.7	1.03 (0.54-1.96)	0.87 (0.46-1.65)	0.87 (0.46-1.65)	0.82 (0.42-1.62)
	Reducer II	6280 (9.1)	32	38 662	0.8	1.28 (0.83-1.97)	1.00 (0.65-1.54)	1.02 (0.66-1.56)	1.01 (0.65-1.57)
	Sustainer	14 693 (21.2)	59	91 292	0.6	1 [Reference]	1 [Reference]	1 [Reference]	1 [Reference]
	Increaser	27 208 (39.3)	114	168 476	0.7	1.05 (0.77-1.44)	1.09 (0.80-1.50)	1.08 (0.79-1.48)	1.10 (0.80-1.52)
Moderate smokers: 10-19 CPD (n = 295 770)	Quitter	47 357 (16.0)	102	298 035	0.3	1.05 (0.84-1.32)	0.93 (0.74-1.17)	0.94 (0.74-1.18)	0.95 (0.75-1.20)
	Reducer I	13 062 (4.4)	49	80 973	0.6	1.86 (1.37-2.52)	1.24 (0.91-1.68)	1.23 (0.90-1.67)	1.14 (0.83-1.56)
	Reducer II	38 056 (12.9)	101	237 973	0.4	1.31 (1.04-1.65)	1.18 (0.93-1.48)	1.19 (0.94-1.49)	1.19 (0.94-1.50)
	Sustainer	125 764 (42.5)	256	788 212	0.3	1 [Reference]	1 [Reference]	1 [Reference]	1 [Reference]
	Increaser	71 531 (24.2)	177	447 233	0.4	1.22 (1.01-1.48)	1.35 (1.11-1.63)	1.32 (1.09-1.60)	1.30 (1.07-1.58)
Heavy smokers: ≥20 CPD (n = 424 470)	Quitter	49 185 (11.6)	99	309 183	0.3	0.94 (0.76-1.17)	0.78 (0.63-0.96)	0.84 (0.67-1.04)	0.83 (0.67-1.04)
	Reducer I	45 011 (10.6)	172	279 395	0.6	1.82 (1.53-2.16)	1.31 (1.10-1.57)	1.32 (1.11-1.57)	1.29 (1.07-1.54)
	Reducer II	67 554 (15.9)	123	423 302	0.3	0.86 (0.71-1.05)	0.99 (0.81-1.20)	1.01 (0.83-1.23)	1.01 (0.83-1.24)
	Sustainer	235 936 (55.6)	499	1 475 778	0.3	1 [Reference]	1 [Reference]	1 [Reference]	1 [Reference]
	Increaser	26 784 (6.3)	51	167 517	0.3	0.90 (0.68-1.20)	1.08 (0.81-1.44)	1.05 (0.78-1.40)	1.02 (0.76-1.37)

Abbreviations: aHR, adjusted hazard ratio; CPD, cigarettes per day; HR, hazard ratio; IR, incidence rate; SHR, subdistribution hazard ratio.

^a^ Quitters were defined as those who stopped smoking; reducers, those who decreased the number of cigarettes smoked per day by 50% or more (reducers I) or by 20% to 50% (reducers II); sustainers, those who maintained (decreased or increased) the number of cigarettes smoked per day by less than 20%; and increasers, those who increased the number of cigarettes smoked per day by 20% or more.

^b^ Calculated by number of participants with follow-up duration.

^c^ Adjusted for age.

^d^ Adjusted for age, sex, income, alcohol consumption, regular physical activity, area of residence, comorbidities (hypertension, diabetes, dyslipidemia, and chronic kidney disease), and body mass index.

### Association Between Change in Smoking Intensity and Risk of Dementia

During the median IQR follow-up period of 6.3 (6.1-6.6) years, there were 11 912 dementia events, including 8800 AD and 1889 VaD events. Table 2 shows the association between relative change in cigarette smoking intensity and risk of dementia. Overall, smoking cessation was associated with an 8% decreased risk of all dementia, a 6% decreased risk of AD, and a 16% decreased risk of VaD, whereas smoking cessation was associated with a 25% increased risk of dementia in the reducer I group and 6% increased risk of dementia in the reducer II group. Those in the quitter group had a lower risk of all dementia (adjusted HR [aHR], 0.92; 95% CI, 0.87-0.97) compared with those in the sustainer group. A higher risk of all dementia was found in the reducer I (aHR, 1.25; 95% CI, 1.18-1.33) and increaser (1.12; 95% CI, 1.06-1.18) groups compared with the sustainer group. However, risk of all dementia among the reducer II group was not significantly different from that in the sustainer group (aHR, 1.06; 95% CI, 1.00-1.12) (eFigure 1 in Supplement 1). These results were in accordance with the association observed in the restricted cubic spline curve (eFigure 2 in Supplement 1). The patterns for AD and VaD remained consistent with the patterns for all dementia.

In the analysis stratified by level of smoking intensity at the 2009 health examination, there was no difference in the risk of all dementia and AD in participants who quit from mild smoking (aHR, 1.00 [95% CI, 0.87-1.15] for all dementia; aHR, 1.08 [95% CI, 0.92-1.25] for AD), but quitting from moderate smoking was associated with a lower risk of dementia (aHR, 0.88; 95% CI, 0.81-0.97). Quitting from heavy smoking was not associated with a lower risk of dementia (aHR, 0.92; 95% CI, 0.85-1.01) (Table 2).

The Figure depicts the association between categorical change in smoking intensity and risk of dementia. Those who smoked persistently regardless of intensity through the 2 health examinations showed 12% to 24% increase in risk of all dementia, including AD and VaD, compared with those who quit. As shown in eTable 3 in Supplement 1, smoking reduction, such as a categorical change from heavy to mild smoking, was not associated with a reduced risk of dementia but was associated with increased risk of all dementia compared with sustained heavy smoking (aHR, 1.33; 95% CI, 1.15-1.54). Results of the sensitivity analysis with competing risk model and multiple imputation were consistent with those in the main analysis (Table 2; eTable 6 in Supplement 1).

**Figure.**
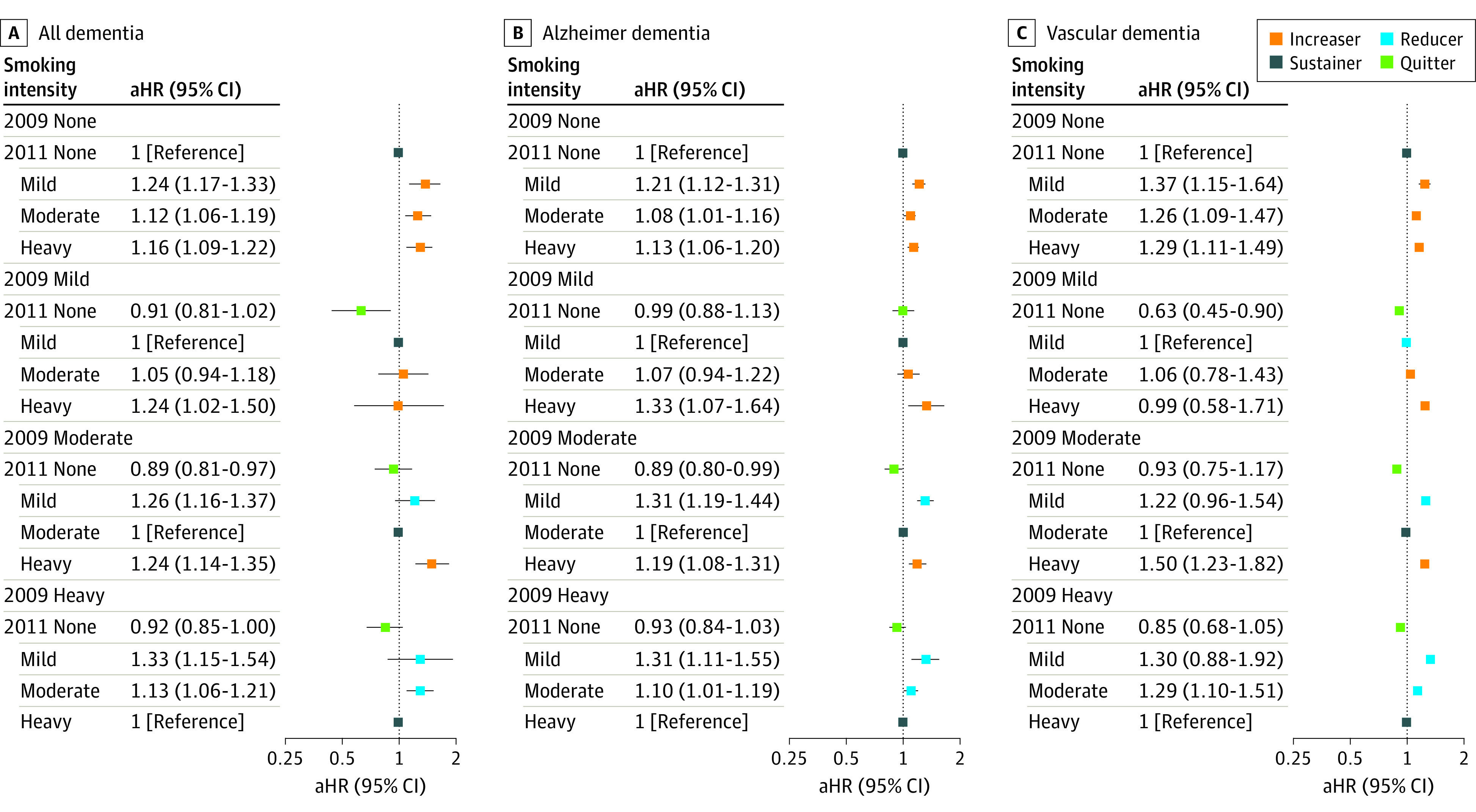
Association Between Categorical Change in Smoking Intensity and Risk of Dementia Quitters were defined as those who stopped smoking; reducers (I and II), those who decreased the number of cigarettes smoked per day by at least 20%; sustainers, those who decreased or increased the number of cigarettes smoked per day by less than 20%; and increasers, those who increased the number of cigarettes smoked per day by at least 20%. The hazard ratio was adjusted for age, sex, household income, alcohol consumption, regular physical activity, area of residence, comorbidities, and body mass index. Error bars represent 95% CIs. aHR indicates adjusted hazard ratio.

### Analysis Stratified by Age, Sex, and Alcohol Consumption

In analyses stratified according to age, sex, and alcohol drinking, the results were consistent with the main findings. The decreased risk of all dementia in participants in the quitter group was more prominent in those younger than 65 years (aHR, 0.81; 95% CI, 0.73-0.90) than in those 65 years or older (aHR, 0.97; 95% CI, 0.91-1.04) (*P* for interaction = 0.003) (eTable 7 in Supplement 1).

There was no significant difference in the association between smoking intensity change and risk of all dementia, including AD and VaD, in males and females, even though no significant results were found in females due to the small number analyzed in the study (eTable 8 in Supplement 1). The decreased risk of all dementia, including AD, in the quitter group was more prominent among alcohol drinkers than nondrinkers (aHR, 0.84 [95% CI, 0.77-0.90] vs 1.02 [95% CI, 0.95-1.11] for all dementia; aHR, 0.82 [95% CI, 0.75-0.90] vs 1.08 [0.99-1.18] for AD; *P *for interaction for all <.001) (eTable 9 in Supplement 1).

## Discussion

To our knowledge, this study was the first to assess the associations between change in smoking intensity and risk of dementia and its subtypes. Smoking cessation was associated with decreased risk (8% decrease for all dementia, 6% for AD, and 16% for VaD) compared with sustained cigarette smoking intensity through the 2 health examinations 2 years apart. Participants who reduced their cigarette use had an increased risk of all dementia: 25% in the reducer I group and 6% in the reducer II group. Findings from the sensitivity analysis support the robustness of these results.

Previous studies^3,4,5,6^ reported that smoking cessation was associated with a decreased risk of dementia, consistent with results of the present study. There are several potential biological mechanisms by which smoking cessation decreases the risk of dementia. Increased cerebral oxidative stress along with decreased antioxidant concentrations due to smoking can contribute to neuropathological alterations, such as amyloid-β (Aβ) aggregation, neuroinflammation, and tau phosphorylation.^28^ A case-control study noted that cigarette smoking is associated with risk biomarkers for AD in cerebrospinal fluid, which indicates excessive oxidative stress, neuroinflammation, and impaired neuroprotection facilitating amyloidogenesis (eg, higher level of tumor necrosis factor α and lower level of brain-derived neurotrophic factor).^29^ Moreover, increased exposure of vascular structures to oxidative stress can induce endothelial cell dysfunction and prothrombotic conditions, which promote cerebral atherosclerosis leading to VaD.^30^ Decreased cerebral blood flow associated with atherosclerosis can accelerate the synthesis of Aβ.^31^ The increased risk of dementia in the increaser group in this study supports these suggested mechanisms.

Although some studies have investigated smoking cessation as a modifiable risk factor for dementia, to our knowledge, the association between smoking reduction and risk of dementia has not been investigated in previous studies. In the present study, we unexpectedly found a 25% increased risk of dementia among those who reduced the number of cigarettes they smoked per day by 50% or more (reducer I group) compared with those who sustained, which was a greater increase than that in the increaser group. One possible explanation for this finding is the sick quitter phenomenon. A reduction or cessation of cigarette smoking could suggest behavioral changes toward a healthy lifestyle because of health concerns, considering the higher number of prevalent comorbidities in the reducer group than the sustainer group. In this context, the decreased risk of dementia associated with smoking cessation could be underestimated because of the sick quitter phenomenon. However, the protective association between smoking cessation and dementia despite the high number of comorbid conditions in those who quit suggests that the sick quitter or reducer phenomenon cannot solely explain the findings. Randomized clinical trials to rule out the sick quitter or reducer phenomenon are not feasible because of ethical issues.

Compensatory smoking among reducers is another potential explanation for the findings. Reducers might inhale deeply to maintain their nicotine levels, negating any potential health benefit.^32^ The increased risk of dementia in the reducer group might partly be explained by deprivation of the cognition-enhancing properties of nicotine. Nicotine is delivered rapidly to the brain and acts at nicotinic acetylcholine receptors (nAChRs) to modulate the release of various neurotransmitters, including acetylcholine, dopamine, and serotonin, at the prefrontal cortex and hippocampus.^33^ Prolonged activation of nAChR by repeated nicotine exposure (eg, cigarette smoking) desensitizes and upregulates nAChR density,^34^ which unmasks cognitive impairment as the amount of nicotine exposure is reduced, resulting in a higher level of unbound nAChRs (eg, smoking reduction). Although there could be poorer memory function after short-term nicotine withdrawal,^35^ prolonged smoking cessation might ameliorate withdrawal-induced cognitive deficits through neuroadaptive recovery.

Analyses stratified by age, sex, and alcohol consumption yielded results that were generally consistent with the main findings. The association of smoking cessation with a decreased risk of dementia was evident in younger participants but not in older age groups. This finding suggests that smoking cessation at a younger age is associated with greater benefits than smoking cessation at an older age. The association between smoking and risk of dementia in older individuals might be underestimated because of the high probability of death (competing mortality) in older individuals. We also did not find a difference by sex in the association between change in smoking intensity and risk of dementia.

The association between smoking cessation and risk of dementia was more prominent among alcohol drinkers than nondrinkers (aHR, 0.84 vs 1.02; *P* for interaction <.001). The combination of smoking and alcohol consumption may be related to this association. A previous study^36^ reported that alcohol consumption was associated with a lower risk of AD among nonsmokers, which suggested an interaction between smoking and alcohol consumption and the risk of AD. In addition, combined smoking and alcohol drinking were associated with a higher risk of AD and VaD than smoking only or drinking only.^37^

The clinical implications of this study are that smoking cessation plays a role in reduced risk of dementia, whereas smoking reduction does not. Smoking reduction might affect health outcomes differently depending on the etiology. For example, smoking reduction has been associated with a lower risk of cancer, in particular lung cancer, but not with a lower risk of cardiovascular diseases.^14,19^ To our knowledge, the association of smoking reduction with dementia has not been examined, although the findings of this study suggest that smoking reduction is not a factor in decreased risk of dementia. This phenomenon may be due to dementia being mainly caused by vascular dysfunction, excessive oxidative stress, and neuronal injury, which do not have a dose-response association with the number of cigarettes smoked, unlike the carcinogenic properties of smoking. Despite the lack of benefits from smoking reduction for risk of dementia, a reduction-to-quit intervention may be an important first step toward smoking cessation.^38^

### Limitations

This study has several limitations that should be considered. First, we were not able to collect information on educational level or apolipoprotein E ε4 level, which may be associated with risk of dementia. In addition, we did not have information on secondhand smoking, use of other combusted tobacco (eg, cigars), or use of noncombusted nicotine products (eg, e-cigarettes or heated tobacco product). However, use of cigar or other nicotine product was minimal during the study period. Second, because dementia progresses insidiously during the early stages, a follow-up duration of 6 years may not be sufficient to fully elucidate the associations between changes in smoking and risk of dementia. Furthermore, the interval used in this study to assess changes in smoking status was relatively short; a longer duration of not smoking may have more benefits for dementia reduction.^3,4,6^

Third, information on duration of smoking cessation was lacking. However, as our study design included only participants with current smoking status in 2009 and quitters in 2011, the duration of cessation did not vary significantly (range, 0-3 years). Fourth, the effect size of smoking cessation was relatively small (aHR, 0.92). However, considering the prevalence of dementia and smoking rate, the public health implication would be still substantial. Fifth, this study could not determine causal inference due to its retrospective design and had restricted generalizability to other ethnicities and females because the study population comprised only Koreans who were mostly male. Despite these limitations, this study reported associations between changes in smoking intensity and risk of dementia in a large general population.

## Conclusions

This cohort study showed that smoking cessation was associated with a reduced risk of all dementia, including AD and VaD, compared with sustained smoking intensity. However, smoking reduction was associated with an increased risk of dementia. Therefore, smoking cessation, not smoking reduction, should be emphasized in efforts to reduce the disease burden of dementia.

## Notes

Supplement 1. **eMethods.** Information on Covariates in the Study **eTable 1.** Previous Studies of the Relationship Between Change in Cigarette Smoking Behavior and Incidence of Dementia **eTable 2.** The Frequent Missing Variables **eTable 3.** Questionnaire on Smoking Status in Korean National Health Screening Examinations **eTable 4.** Association Between Categorical Change in Cigarette Smoking Intensity and Risk of Dementia **eTable 5.** Baseline Characteristics of the Study Population Between Inclusion and Exclusion Group **eTable 6.** Association Between Relative Changes in Cigarette Smoking Intensity and Risk of Dementia With Multiple Imputation **eTable 7.** Association Between Relative Change in Cigarette Smoking Intensity and Risk of Dementia Stratified by Age **eTable 8.** Association Between Relative Changes in Cigarette Smoking Intensity and Risk of Dementia Stratified by Sex **eTable 9.** Association Between Relative Changes in Cigarette Smoking Intensity and Risk of Dementia Stratified by Alcohol Drinking Status **eFigure 1.** Graphical Abstract of the Study **eFigure 2.** Restricted Cubic Spline Curve for Association Between Changes in Smoking Intensity With Risk of Dementia

Supplement 2.
Data Sharing Statement
