# Errors in the Byline and Figure

**DOI:** 10.1001/jamanetworkopen.2023.2452

**Published:** 2023-02-27

In the Original Investigation titled “Association of Changes in Smoking Intensity With Risk of Dementia in Korea” published January 19, 2023, there were errors in the byline and in the Figure. The correct names and degrees for 2 coauthors should be Jinkook Lee, PhD and SangYun Kim, MD, PhD. In the Figure, the plots for the first panel and third panel were switched. The missing lines for the 95% CIs have also been added to the first panel. This article has been corrected.^1^
