# Supplementary material for: Association of Changes in Smoking Intensity With Risk of Dementia in Korea
Source: JAMA Netw Open. 2023 Jan 19;6(1):e2251506. doi: 10.1001/jamanetworkopen.2022.51506 (PMC9857334; doi:10.1001/jamanetworkopen.2022.51506)
Supplement: Supplement 1. — eMethods. Information on Covariates in the Study eTable 1. Previous Studies of the Relationship Between Change in Cigarette Smoking Behavior and Incidence of Dementia eTable 2. The Frequent Missing Variables eTable 3. Questionnaire on Smoking Status in Korean National Health Screening Examinations eTable 4. Association Between Categorical Change in Cigarette Smoking Intensity and Risk of Dementia eTable 5. Baseline Characteristics of the Study Population Between Inclusion and Exclusion Group eTable 6. Association Between Relative Changes in Cigarette Smoking Intensity and Risk of Dementia With Multiple Imputation eTable 7. Association Between Relative Change in Cigarette Smoking Intensity and Risk of Dementia Stratified by Age eTable 8. Association Between Relative Changes in Cigarette Smoking Intensity and Risk of Dementia Stratified by Sex eTable 9. Association Between Relative Changes in Cigarette Smoking Intensity and Risk of Dementia Stratified by Alcohol Drinking Status eFigure 1. Graphical Abstract of the Study eFigure 2. Restricted Cubic Spline Curve for Association Between Changes in Smoking Intensity With Risk of Dementia [file jamanetwopen-e2251506-s001.pdf]

Supplemental Online Content

Jeong SM, Park J, Han K, et al. Association of changes in smoking intensity with risk of dementia in Korea. *JAMA Netw Open*. 2023;6(1):e2251506. doi:10.1001/jamanetworkopen.2022.51506

eMethods. Information on Covariates in the Study

eTable 1. Previous Studies of the Relationship Between Change in Cigarette Smoking Behavior and Incidence of Dementia

eTable 2. The Frequent Missing Variables

eTable 3. Questionnaire on Smoking Status in Korean National Health Screening Examinations

eTable 4. Association Between Categorical Change in Cigarette Smoking Intensity and Risk of Dementia

eTable 5. Baseline Characteristics of the Study Population Between Inclusion and Exclusion Group

eTable 6. Association Between Relative Changes in Cigarette Smoking Intensity and Risk of Dementia With Multiple Imputation

eTable 7. Association Between Relative Change in Cigarette Smoking Intensity and Risk of Dementia Stratified by Age

eTable 8. Association Between Relative Changes in Cigarette Smoking Intensity and Risk of Dementia Stratified by Sex

eTable 9. Association Between Relative Changes in Cigarette Smoking Intensity and Risk of Dementia Stratified by Alcohol Drinking Status

eFigure 1. Graphical Abstract of the Study

eFigure 2. Restricted Cubic Spline Curve for Association Between Changes in Smoking Intensity With Risk of Dementia

This supplementary material has been provided by the authors to give readers additional information about their work.

© 2023 Jeong S-M et al. *JAMA Network Open.*

eMethods. Information on Covariates in the Study

Information on covariates was assessed on the day of the second examination in 2011, which served as baseline. Alcohol consumption was classified into four levels: none, mild (<15g/day), moderate (15-30g/day), and heavy (≥30g/day). Daily alcohol intake was calculated by multiplying the average frequency of alcohol intake (per week) and the typical number of standard drinks on each occasion.[1] Regular exercise was defined as more than 30 minutes of moderate physical activity at least five times per week or more than 20 minutes of strenuous physical activity at least three times per week.[2] Body mass index was calculated as weight (kg) divided by height in meters squared (m^2).

Comorbidities were defined using medical claims data before screening according to International Classification of Diseases (ICD-10) codes and relevant medications: for hypertension, I10-I13 or I15 and antihypertensive drugs or blood pressure ≥140/90 mmHg; for diabetes, E11-E14 and anti-diabetic drugs or fasting glucose level ≥126 mg/dL; for dyslipidemia, E78 + lipid lowering drugs or total cholesterol level ≥240 mg/dL. Chronic kidney disease was defined as glomerular filtration rate <60 ml/min/1.73m^2 as estimated by the Modification of Diet in Renal Disease equation. Household income was categorized into four quartiles based on insurance premium level. In Korea, insurance premium levels are determined based on income. Medical aid recipients (the poorest 3% of the population) were merged into the lowest income quintile.

Reference

1. Yoo JE, Shin DW, Han K, et al. Association of the Frequency and Quantity of Alcohol Consumption With Gastrointestinal Cancer. *JAMA Netw Open*. Aug 2 2021;4(8):e2120382. doi:10.1001/jamanetworkopen.2021.20382
2. Anton SD, Duncan GE, Limacher MC, Martin AD, Perri MG. How much walking is needed to improve cardiorespiratory fitness? An examination of the 2008 Physical Activity Guidelines for Americans. *Res Q Exerc Sport*. Jun 2011;82(2):365-70. doi:10.1080/02701367.2011.10599766

© 2023 Jeong S-M et al. *JAMA Network Open.*

eTable 1. Previous Studies of the Relationship Between Change in Cigarette Smoking Behavior and the Incidence of Dementia

Author (year)	Study design /country	Number of subjects	Age range (years)	Follow-up (years)	Adjustment	Outcomes	Number of cases	Smoking assessment	Group	Result [HR (95%CI)]
With two times of separate smoking cessation assessment										
Choi et al (2018)	Retrospective cohort study<br><br>/Korea	46,140	≥ 60 (2002-2013)	8 (mean years 7.11)	Age, BMI, BP, Fasting glucose, total cholesterol, physical activity, drinking habit, household income, Charlson Comorbidity Index	Dementia (ICD-10 codes F00-F03, G30-G32)	1,644	Questionnaire from both the first and second health examinations.	Continual smoker<br>Short-term quitter (quit <4years)<br>Long-term quitter (quit ≥4years)<br>Never smoker	1.00 (ref)<br>0.87 (0.72–1.05)<br><br>0.86 (0.75–0.99)<br><br>0.81 (0.71–0.91)
Deal et al (2020)	Prospective cohort study (ARIC study)<br><br>/USA	13,002	45-64 (from 1987-1989 to 2011-2013)	Up to 12 (5 year lag)	Age, sex, education, a combination variable of race and study site, BMI, history of stroke, diabetes, hypertension and COPD.	Dementia (standardized algorithms confirmed by expert panel review at the visit or Modified TICS-modified interview on modified CDR)	1,347	Self-reported cigarette smoking status during annual follow-up telephone calls from visit 1 (1987-89) and visit 4 (1996-98)	Current smoker<br>Former smoker (quit< 4years ago)<br>Former smoker (quit ≥9years ago)<br>Never smoker	1.40 (1.02–1.92)<br>1.34 (0.91–1.99)<br><br>1.13 (0.91–1.39)<br><br>1.00 (ref)
Based on self-reported smoking history										
Lu et al (2020)	Cohort study (Ohsaki Cohort 2006 study)<br><br>/Japan	12,489	≥ 65 (2006-2007)	5.7	BMI, time spent walking, alcohol drinking status, history of diseases (stroke, myocardial infarction, hypertension or diabetes) education level, psychological distress, cognitive function (kihon checklist)	Dementia (LTCI system criteria)	1,110	Questionnaire at the baseline	Current smoker<br>Ex-smoker<br>(≤2 years<br>3-5 years<br>6-10 years<br>11-15 years<br>>15 years)<br>Never smoker	1.46 (1.17–1.80)<br><br>1.39 (0.96–2.01)<br>1.03 (0.70–1.53)<br>1.04 (0.74–1.45)<br>1.19 (0.84–1.69)<br>0.92 (0.73–1.15)<br>1.00 (ref)
Johnson et al (2021)	Cohort study (from NACC UDS)<br><br>/USA	33,444	≥ 45 (2005-2018)	14	Sex, education, diabetes status, hypertension status.	Dementia (clinical evaluation, CDR global score ≥ 1)	806	Interview at visit	Never smoker<br>Former smoker<br>Current smoker<br><br>Never smoker<br>Quit < 10years<br>Quit 10 to <20years<br>Quit 20 to <30years<br>Quit ≥ 30years	1.000 (ref)<br>0.845 (0.730–0.978)<br>1.656 (1.181–2.321)<br><br>1.000 (ref)<br>1.171 (0.728–1.885)<br>1.206 (0.883–1.646)<br>0.790 (0.597-1.046)<br>0.780 (0.655-0.930)

© 2023 Jeong S-M et al. *JAMA Network Open.*

AD, Alzheimer’s disease; VaD, Vascular Dementia; DSM, Diagnostic and Statistical Manual of Mental Disorders; RR, risk ratio; HR, hazard ratio; OR, odds ratio; BP, blood pressure; BMI, body mass index; COPD, chronic obstructive pulmonary disease; LTCI, long-term care insurance; TICS, telephone interview for cognitive status; CDR, clinical dementia rating. Statistically significant values are marked in bold.

© 2023 Jeong S-M et al. *JAMA Network Open*.

eTable 2. The Frequent Missing Variables

Variables	Missing (%)
Age	0.01
Sex	0.01
Residence place	0.02
smoking status in 2009	16.04
Alcohol consumption	2.06
Regular physical activity	1.02
Income	19.11
Diabetes mellitus	0.07
Hypertension	0.06
Dyslipidemia	0.06
Chronic kidney disease	0.08
Body mass index	0.05
Weight	0.05
Height	0.05
Waist circumference	0.12
Systolic blood pressure	0.07
Diastolic blood pressure	0.07
Glucose	0.08
Total cholesterol	0.07
HDL-C	0.07
LDL-C	14.0
GFR	0.08

HDL, high-density lipoprotein-cholesterol; LDL, low-density lipoprotein-cholesterol; GFR, glomerular filtration rate.

© 2023 Jeong S-M et al. *JAMA Network Open.*

eTable 3. Questionnaire on Smoking Status in Korean National Health Screening Examinations

1-1. Have you smoked at least 100 cigarettes in your entire life?
a. No<br>b. Yes, I used to smoke but I stopped ☞ (Go to the question 1-2)<br>c. Yes, I'm still smoking ☞ (Go to the question 1-3)
1-2. If you used to smoke but stopped, please answer the following.,<br>How long had you smoked before quitting? Total ______ years<br>At that time, how many cigarettes did you usually smoke per day? ______ cigarettes per day
1-3. If you are still smoking, please answer the following.<br>How long have you been smoking? Total ______ years<br>How many cigarettes do you usually smoke per day? ______ cigarettes per day

© 2023 Jeong S-M et al. *JAMA Network Open.*

eTable 4. Association Between Categorical Changes in Cigarette Smoking Intensity and Risk of Dementia

Smoking Status 2009	Smoking Status 2011	No. (%)	Case No.	Duration	IR	Crude model HR (95% CI)	Age-adjusted model aHR[a] (95% CI)	Multivariate model aHR[b] (95% CI)
All Dementia (Total)								
All current smokers (N=789,532)	Non (quitter)	114,959 (14.6)	1,730	722,407	2.4	1 (Ref.)	1 (Ref.)	1 (Ref.)
	Mild	60,417 (7.7)	2,056	374,027	5.5	2.30 (2.16-2.45)	1.33 (1.25-1.42)	1.24 (1.17-1.33)
	Moderate	262,132 (33.2)	3,866	1,640,146	2.4	0.99 (0.93-1.05)	1.17 (1.10-1.23)	1.12 (1.06-1.19)
	Heavy	352,024 (44.6)	4,260	2,201,224	1.9	0.81 (0.77-0.86)	1.23 (1.16-1.30)	1.16 (1.09-1.22)
Mild smokers, <10 CPD (N=69,292)	Non (quitter)	18,417 (26.6)	435	115,189	3.8	0.67 (0.60-0.75)	0.88 (0.79-0.98)	0.91 (0.81-1.02)
	Mild	31,428 (45.4)	1095	194,452	5.6	1 (Ref.)	1 (Ref.)	1 (Ref.)
	Moderate	15,387 (22.2)	432	95,463	4.5	0.80 (0.72-0.90)	1.05 (0.94-1.17)	1.05 (0.94-1.18)
	Heavy	4,060 (5.9)	120	25,101	4.8	0.85 (0.70-1.03)	1.25 (1.03-1.51)	1.24 (1.02-1.50)
Moderate smokers, 10-19 CPD (N=295,770)	Non (quitter)	47,357 (16.0)	637	298,035	2.1	1.04 (0.95-1.13)	0.85 (0.78-0.93)	0.89 (0.81-0.97)
	Mild	22,635 (7.7)	764	140,252	5.4	2.65 (2.44-2.87)	1.30 (1.20-1.41)	1.26 (1.16-1.37)
	Moderate	174,194 (58.9)	2,242	1,092,041	2.1	1 (Ref.)	1 (Ref.)	1 (Ref.)
	Heavy	51,584 (17.4)	773	322,098	2.4	1.17 (1.08-1.27)	1.26 (1.16-1.37)	1.24 (1.14-1.35)
Heavy Smokers, ≥ 20 CPD (N=424,470)	Non (quitter)	49,185 (11.6)	658	309,183	2.1	1.16 (1.07-1.27)	0.86 (0.79-0.93)	0.92 (0.85-1.01)
	Mild	6,354 (1.5)	197	39,324	5.0	2.74 (2.38-3.17)	1.30 (1.12-1.50)	1.33 (1.15-1.54)
	Moderate	72,551 (17.1)	1,192	452,642	2.6	1.45 (1.36-1.55)	1.11 (1.04-1.19)	1.13 (1.06-1.21)
	Heavy	296,380 (69.8)	3,367	1,854,025	1.8	1 (Ref.)	1 (Ref.)	1 (Ref.)
Alzheimer's dementia								
All current smokers (N=789,532)	Non (quitter)	114,959 (14.6)	1,314	722,407	1.8	1 (Ref.)	1 (Ref.)	1 (Ref.)
	Mild	60,417 (7.7)	1,599	374,027	4.3	2.35 (2.19-2.53)	1.30 (1.21-1.40)	1.21 (1.12-1.30)
	Moderate	262,132 (33.2)	2,836	1,640,146	1.7	0.96 (0.90-1.02)	1.13 (1.06-1.21)	1.08 (1.01-1.16)
	Heavy	352,024 (44.6)	3,051	2,201,224	1.4	0.77 (0.72-0.82)	1.20 (1.12-1.28)	1.13 (1.06-1.20)
Mild smokers, <10 CPD (N=69,292)	Non (quitter)	18,417 (26.6)	357	115,189	3.1	0.71 (0.63-0.81)	0.95 (0.84-1.08)	1.00 (0.88-1.13)
	Mild	31,428 (45.4)	842	194,452	4.3	1 (Ref.)	1 (Ref.)	1 (Ref.)
	Moderate	15,387 (22.2)	332	95,463	3.5	0.80 (0.71-0.91)	1.07 (0.94-1.21)	1.07 (0.94-1.22)
	Heavy	4,060 (5.9)	95	25,101	3.8	0.88 (0.71-1.08)	1.33 (1.07-1.64)	1.33 (1.07-1.64)
Moderate smokers, 10-19 CPD (N=295,770)	Non (quitter)	47,357 (16.0)	469	298,035	1.6	1.04 (0.94-1.15)	0.85 (0.77-0.94)	0.89 (0.80-0.98)
	Mild	22,635 (7.7)	607	140,252	4.3	2.86 (2.60-3.13)	1.35 (1.23-1.48)	1.31 (1.19-1.44)
	Moderate	174,194 (58.9)	1,649	1,092,041	1.5	1 (Ref.)	1 (Ref.)	1 (Ref.)
	Heavy	51,584 (17.4)	540	322,098	1.7	1.11 (1.01-1.23)	1.21 (1.10-1.34)	1.19 (1.08-1.31)
Heavy Smokers, ≥ 20 CPD (N=424,470)	Non (quitter)	49,185 (11.6)	488	309,183	1.6	1.20 (1.09-1.33)	0.87 (0.79-0.96)	0.93 (0.84-1.03)
	Mild	6,354 (1.5)	150	39,324	3.8	2.91 (2.46-3.43)	1.28 (1.08-1.51)	1.31 (1.11-1.55)
	Moderate	72,551 (17.1)	855	452,642	1.9	1.45 (1.34-1.57)	1.08 (1.00-1.16)	1.10 (1.01-1.19)
	Heavy	296,380 (69.8)	2,416	1,854,025	1.3	1 (Ref.)	1 (Ref.)	1 (Ref.)
Vascular dementia								
	Non (quitter)	114,959 (14.6)	245	722,407	0.3	1 (Ref.)	1 (Ref.)	1 (Ref.)

© 2023 Jeong S-M et al. *JAMA Network Open.*

All current smokers (N=789,532)	Mild	60,417 (7.7)	259	374,027	0.7	2.05 (1.72-2.44)	1.42 (1.19-1.69)	1.37 (1.15-1.64)
	Moderate	262,132 (33.2)	620	1,640,146	0.4	1.12 (0.96-1.30)	1.30 (1.12-1.50)	1.26 (1.09-1.47)
	Heavy	352,024 (44.6)	765	2,201,224	0.3	1.03 (0.89-1.19)	1.36 (1.18-1.58)	1.29 (1.11-1.49)
Mild smokers, <10 CPD (N=69,292)	Non (quitter)	18,417 (26.6)	44	115,189	0.4	0.53 (0.38-0.74)	0.64 (0.46-0.90)	0.64 (0.45-0.90)
	Mild	31,428 (45.4)	140	194,452	0.7	1 (Ref.)	1 (Ref.)	1 (Ref.)
	Moderate	15,387 (22.2)	61	95,463	0.6	0.89 (0.66-1.20)	1.08 (0.80-1.46)	1.06 (0.78-1.43)
	Heavy	4,060 (5.9)	15	25,101	0.6	0.83 (0.49-1.42)	1.07 (0.62-1.82)	1.00 (0.58-1.71)
Moderate smokers, 10-19 CPD (N=295,770)	Non (quitter)	47,357 (16.0)	102	298,035	0.3	1.09 (0.87-1.36)	0.93 (0.74-1.15)	0.93 (0.75-1.17)
	Mild	22,635 (7.7)	92	140,252	0.7	2.09 (1.66-2.63)	1.23 (0.97-1.55)	1.22 (0.96-1.54)
	Moderate	174,194 (58.9)	342	1,092,041	0.3	1 (Ref.)	1 (Ref.)	1 (Ref.)
	Heavy	51,584 (17.4)	149	322,098	0.5	1.48 (1.22-1.79)	1.53 (1.26-1.86)	1.50 (1.23-1.82)
Heavy Smokers, ≥ 20 CPD (N=424,470)	Non (quitter)	49,185 (11.6)	99	309,183	0.3	0.98 (0.80-1.22)	0.78 (0.63-0.97)	0.85 (0.68-1.05)
	Mild	6,354 (1.5)	27	39,324	0.7	2.11 (1.44-3.11)	1.28 (0.87-1.88)	1.30 (0.88-1.92)
	Moderate	72,551 (17.1)	217	452,642	0.5	1.48 (1.27-1.73)	1.26 (1.08-1.47)	1.29 (1.10-1.51)
	Heavy	296,380 (69.8)	601	1,854,025	0.3	1 (Ref.)	1 (Ref.)	1 (Ref.)

IR, incidence rate; HR, hazard ratio; aHR, adjusted hazard ratio; CI, confidence interval; CPD, cigarettes per day.

[a]Adjusted for age.

[b]Adjusted for age, sex, income, alcohol, regular exercise, residence, hypertension, diabetes mellitus, dyslipidemia, chronic kidney disease, and body mass index.

© 2023 Jeong S-M et al. *JAMA Network Open.*

eTable 5. Baseline Characteristics of the Study Population Between Inclusion and Exclusion Group

Variable (SD)	Inclusion (N = 789,532)	Exclusion (N = 117,739)	*p*-value
Mean age, years	52.2 ± 8.5	52.9 ± 9.0	< 0.001
Sex			
Male, No. (%)	756,469 (95.8)	104,269 (88.6)	< 0.001
Alcohol consumption, No. (%)			
Non	203,562 (25.8)	46,382 (40.2)	< 0.001
Mild	278,957 (35.3)	33,959 (29.4)	
Moderate	178,391 (22.6)	20,136 (17.5)	
Heavy	128,622 (16.3)	14,837 (12.9)	
Regular physical activity, No. (%)			
Non	632,538 (80.1)	92,039 (79.0)	< 0.001
Vigorous or Moderate	119,160 (15.1)	18,507 (15.9)	
Vigorous and Moderate	37,834 (4.8)	5,987 (5.1)	
Anthropometrics			
Height, cm	168.4 ± 6.4	167.3 ± 7.3	< 0.001
Weight, kg	68.0 ± 10.2	68.0 ± 10.7	< 0.001
Waist Circumference, cm	83.5 ± 7.7	83.7 ± 7.9	< 0.001
Body mass index, kg/m^2	23.9 ± 3.0	24.2 ± 3.0	< 0.001
Systolic Blood Pressure, mmHg	124.4 ± 14.3	125.0 ± 14.6	< 0.001
Diastolic Blood Pressure, mmHg	78.2 ± 9.8	78.4 ± 9.9	< 0.001
Comorbidity			
Hypertension, No (%)	241,806 (30.6)	17,529 (14.9)	< 0.001
Diabetes Mellitus, No (%)	103,139 (13.1)	38,206 (32.5)	< 0.001
Dyslipidemia, No (%)	160,062 (20.3)	28,891 (24.6)	< 0.001
Chronic Kidney Disease, No (%)	31,577 (4.0)	5,278 (4.5)	< 0.001
Laboratory findings			
Glucose, mg/dL	102.2 ± 27.6	104.7 ± 31.0	< 0.001
Cholesterol, mg/dL	198.2 ± 36.1	202.9 ± 40.5	< 0.001
HDL, mg/dL	52.1 ± 16.8	51.5 ± 17.5	< 0.001
LDL, mg/dL	114.5 ± 35.3	115.6 ± 36.3	< 0.001
GFR, mL/min/1.73m^2	88.9 ± 37.4	88.2 ± 32.2	< 0.001
Urban residency, No. (%)	355,838 (45.1)	51,151 (43.5)	< 0.001
Household Income, No (%)			
Q1(lowest)	147,697 (18.7)	16,183 (17.0)	< 0.001
Q2	134,155 (17.0)	16,244 (17.1)	
Q3	213,459 (27.0)	23,422 (24.6)	
Q4(highest)	294,221 (37.3)	39,390 (41.4)	
Smoking status in 2009, No. (%)			
Mild, <10 cigarettes/day	69,292 (8.8)	15,753 (15.9)	< 0.001
Moderate, 10-19 cigarettes/day	295,770 (37.5)	36,226 (36.6)	
Heavy, ≥20 cigarettes/day	424,470 (53.8)	46,873 (47.4)	
Duration of smoking in 2009, No. (%)			
<5 y	18,674 (2.4)	6,248 (6.3)	< 0.001
5-9 y	18,229 (2.3)	4,207 (4.2)	
10-19 y	119,613 (15.2)	17,628 (17.8)	
20-29 y	361,970 (45.9)	39,456 (39.8)	
≥30 y	271,046 (34.3)	31,614 (31.9)	
PY of smoking in 2009, No. (%)			
<10 PY	129,430 (16.4)	23,507 (25.4)	< 0.001
10 to <20 PY	227,456 (28.8)	24,562 (26.6)	
20 to <30 PY	222,228 (28.2)	22,724 (24.6)	
≥30 PY	210,418 (26.7)	21,663 (23.4)	
Smoking status in 2011, No. (%)			
Non, 0 cigarette/day	114,959 (14.6)	10,220 (18.9)	< 0.001
Mild, <10 cigarettes/day	60,417 (7.7)	3,930 (7.3)	
Moderate, 10-19 cigarettes/day	262,132 (33.2)	16,960 (31.3)	
Heavy, ≥20 cigarettes/day	352,024 (44.6)	23,023 (42.5)	

© 2023 Jeong S-M et al. *JAMA Network Open.*

Duration of smoking in 2011, No. (%)			
<5 y	125,034 (15.8)	1,694 (3.2)	< 0.001
5-9 y	13,652 (1.7)	1,043 (2.0)	
10-19 y	83,989 (10.6)	6,687 (12.7)	
20-29 y	294,061 (37.2)	23,634 (45.0)	
≥30 y	272,796 (34.6)	19,464 (37.1)	
PY of smoking in 2011, No. (%)			
<10 PY	124,411 (15.8)	10,797 (17.5)	< 0.001
10 to <20 PY	224,677 (28.5)	17,600 (28.5)	
20 to <30 PY	214,052 (27.1)	16,977 (27.5)	
≥30 PY	226,392 (28.7)	16,462 (26.6)	

SD, standard deviation; HDL, high-density lipoprotein; LDL, low-density lipoprotein; GFR, glomerular filtration rate; PY, pack-years All variables were assess in second health exam (2011) except for smoking status of first health exam (2009)

© 2023 Jeong S-M et al. *JAMA Network Open.*

eTable 6. Association Between Relative Changes in Cigarette Smoking Intensity and Risk of Dementia With Multiple Imputation

Smoking Status		Multiple Imputation
2009	2011	
All Dementia (Total)		
All current smokers (N=789,532)	Quitter	0.90 (0.85-0.96)
	Reducer I	1.21 (1.13-1.29)
	Reducer II	1.04 (0.98-1.10)
	Sustainer	1 (Ref.)
	Increaser	1.10 (1.05-1.15)
Mild smokers, <10 CPD (N=69,292)	Quitter	0.95 (0.84-1.08)
	Reducer I	1.12 (0.86-1.48)
	Reducer II	1.11 (0.98-1.25)
	Sustainer	1 (Ref.)
	Increaser	1.12 (1.00-1.24)
Moderate smokers, 10-19 CPD (N=295,770)	Quitter	0.88 (0.80-0.96)
	Reducer I	1.28 (1.15-1.43)
	Reducer II	1.08 (0.99-1.17)
	Sustainer	1 (Ref.)
	Increaser	1.15 (1.06-1.24)
Heavy Smokers, ≥ 20 CPD (N=424,470)	Quitter	0.90 (0.83-0.99)
	Reducer I	1.19 (1.10-1.27)
	Reducer II	0.99 (0.91-1.08)
	Sustainer	1 (Ref.)
	Increaser	1.05 (0.94-1.18)
Alzheimer’s dementia		
All current smokers (N=789,532)	Quitter	0.93 (0.87-0.99)
	Reducer I	1.20 (1.12-1.30)
	Reducer II	1.03 (0.97-1.10)
	Sustainer	1 (Ref.)
	Increaser	1.07 (1.01-1.14)
Mild smokers, <10 CPD (N=69,292)	Quitter	1.02 (0.89-1.18)
	Reducer I	1.15 (0.88-1.50)
	Reducer II	1.10 (0.97-1.28)
	Sustainer	1 (Ref.)
	Increaser	1.09 (0.96-1.22)
Moderate smokers, 10-19 CPD (N=295,770)	Quitter	0.88 (0.79-0.98)
	Reducer I	1.32 (1.18-1.48)
	Reducer II	1.07 (0.97-1.19)
	Sustainer	1 (Ref.)
	Increaser	1.12 (1.02-1.23)
Heavy Smokers, ≥ 20 CPD (N=424,470)	Quitter	0.91 (0.82-1.01)
	Reducer I	1.15 (1.06-1.25)
	Reducer II	0.99 (0.90-1.09)
	Sustainer	1 (Ref.)
	Increaser	1.05 (0.91-1.22)
Vascular dementia		
All current smokers (N=789,532)	Quitter	0.82 (0.71-0.94)
	Reducer I	1.18 (1.03-1.36)
	Reducer II	1.07 (0.94-1.21)
	Sustainer	1 (Ref.)
	Increaser	1.15 (1.02-1.29)
Mild smokers, <10 CPD (N=69,292)	Quitter	0.65 (0.44-0.96)
	Reducer I	0.79 (0.41-1.51)
	Reducer II	0.95 (0.65-1.39)
	Sustainer	1 (Ref.)
	Increaser	1.09 (0.82-1.44)

© 2023 Jeong S-M et al. *JAMA Network Open.*

Moderate smokers, 10-19 CPD (N=295,770)	Quitter	0.89 (0.71-1.12)
	Reducer I	1.14 (0.79-1.64)
	Reducer II	1.14 (0.92-1.41)
	Sustainer	1 (Ref.)
	Increaser	1.25 (1.03-1.51)
Heavy Smokers, ≥ 20 CPD (N=424,470)	Quitter	0.82 (0.66-1.02)
	Reducer I	1.27 (1.07-1.50)
	Reducer II	1.05 (0.86-1.27)
	Sustainer	1 (Ref.)
	Increaser	1.00 (0.77-1.29)

Adjusted for age, sex, income, alcohol, regular exercise, residence, hypertension, diabetes mellitus, dyslipidemia, chronic kidney disease, and body mass index.

Reducer I (≥50% reduction), reducer II (20-50% reduction), sustainer sustainers (less than 20% reduction or less than 20% increase), or increaser (≥20% increase) from baseline cigarette per day.

© 2023 Jeong S-M et al. *JAMA Network Open.*

eTable 7. Association Between Relative Changes in Cigarette Smoking Intensity and Risk of Dementia Stratified by Age

Subgroup	Smoking status				Sustained smoking at same level as a reference				
	2009	2011	No. (%)		Case No.	Duration	IR	aHR (95% CI)	p for interaction
All Dementia (Total)									
Age (<65)	All Smokers (N=709,933)	Quitter	100,843 (14.2)		443	638,949	0.7	0.81 (0.73-0.90)	0.003
		Reducer I	51,720 (7.3)		410	325,317	1.3	1.33 (1.19-1.48)	
		Reducer II	101,743 (14.3)		509	640,697	0.8	1.02 (0.93-1.13)	
		Sustainer	342,440 (48.2)		1,748	2,156,155	0.8	1 (Ref.)	
		Increaser	113,187 (15.9)		643	712,077	0.9	1.12 (1.03-1.23)	
Age (≥65)	All Smokers (N=79,599)	Quitter	14,116 (17.7)		1,287	83,458	15.4	0.97 (0.91-1.04)	
		Reducer I	9,047 (11.4)		1,175	51,637	22.8	1.25 (1.16-1.33)	
		Reducer II	10,147 (12.7)		1,061	59,240	17.9	1.09 (1.02-1.17)	
		Sustainer	33,953 (42.7)		3,210	199,127	16.1	1 (Ref.)	
		Increaser	12,336 (15.5)		1,426	71,148	20.0	1.13 (1.06-1.20)	
Alzheimer’s dementia									
Age (<65)	All Smokers (N=709,933)	Quitter	100,843 (14.2)		281	638,949	0.4	0.80 (0.70-0.92	0.003
		Reducer I	51,720 (7.3)		271	325,317	0.8	1.35 (1.19-1.55)	
		Reducer II	101,743 (14.3)		321	640,697	0.5	1.02 (0.90-1.16)	
		Sustainer	342,440 (48.2)		1,107	2,156,155	0.5	1 (Ref.)	
		Increaser	113,187 (15.9)		386	712,077	0.5	1.06 (0.95-1.19)	
Age (≥65)	All Smokers (N=79,599)	Quitter	14,116 (17.7)		1,033	83,458	12.4	0.99 (0.92,1.06)	
		Reducer I	9,047 (11.4)		920	51,637	17.8	1.23 (1.14-1.33)	
		Reducer II	10,147 (12.7)		841	59,240	14.2	1.09 (1.01-1.18)	
		Sustainer	33,953 (42.7)		2,529	199,127	12.7	1 (Ref.)	
		Increaser	12,336 (15.5)		1,111	71,148	15.6	1.11 (1.04-1.19)	
Vascular dementia									
Age (<65)	All Smokers (N=709,933)	Quitter	100,843 (14.2)		111	638,949	0.2	0.82 (0.66-1.01)	0.955
		Reducer I	51,720 (7.3)		97	325,317	0.3	1.34 (1.08-1.68)	
		Reducer II	101,743 (14.3)		137	640,697	0.2	1.10 (0.91-1.33)	
		Sustainer	342,440 (48.2)		434	2,156,155	0.2	1 (Ref.)	
		Increaser	113,187 (15.9)		166	712,077	0.2	1.17 (0.98-1.40)	
Age (≥65)	All Smokers (N=79,599)	Quitter	14,116 (17.7)		134	83,458	1.6	0.86 (0.71-1.05)	
		Reducer I	9,047 (11.4)		135	51,637	2.6	1.25 (1.03-1.52)	
		Reducer II	10,147 (12.7)		119	59,240	2.0	1.05 (0.86-1.29)	
		Sustainer	33,953 (42.7)		380	199,127	1.9	1 (Ref.)	

© 2023 Jeong S-M et al. *JAMA Network Open.*

		Increaser	12,336 (15.5)		176	71,148	2.5	1.21 (1.01-1.45)	

IR, incidence rate; aHR, adjusted hazard ratio; CI, confidence interval.
Adjusted for age, sex, income, alcohol, regular exercise, residence, hypertension, diabetes mellitus, dyslipidemia, chronic kidney disease, and body mass index.
Reducer I (≥50% reduction), reducer II (20-50% reduction), sustainer sustainers (less than 20% reduction or less than 20% increase), or increaser (≥20% increase) from baseline cigarette per day.

© 2023 Jeong S-M et al. *JAMA Network Open.*

eTable 8. Association Between Relative Changes in Cigarette Smoking Intensity and Risk of Dementia Stratified by Sex

Subgroup	Smoking status				Sustained smoking at same level as a reference				
	2009	2011	No. (%)		Case No.	Duration	IR	aHR (95% CI)	p for interaction
All Dementia (Total)									
Male	All Smokers (N=756,469)	Quitter	110,148 (14.6)		1,614	692,130	2.3	0.92 (0.87-0.98)	0.357
		Reducer I	56,695 (7.5)		1,396	351,705	4.0	1.26 (1.19-1.34)	
		Reducer II	107,389 (14.2)		1,403	671,823	2.1	1.04 (0.98-1.11)	
		Sustainer	363,628 (48.1)		4,560	2,275,321	2.0	1 (Ref.)	
		Increaser	118,609 (15.7)		1,800	740,071	2.4	1.11 (1.06-1.18)	
Female	All Smokers (N=33,063)	Quitter	4,811 (14.6)		116	30,277	3.8	0.86 (0.69-1.05)	
		Reducer I	4,072 (12.3)		189	25,249	7.5	1.17 (0.98-1.39)	
		Reducer II	4,501 (13.6)		167	28,114	5.9	1.17 (0.98-1.40)	
		Sustainer	12,765 (38.6)		398	79,961	5.0	1 (Ref.)	
		Increaser	6,914 (20.9)		269	43,154	6.2	1.12 (0.96-1.30)	
Alzheimer's dementia									
Male	All Smokers (N=756,469)	Quitter	110,148 (14.6)		1,220	692,130	1.8	0.94 (0.88-1.01)	0.247
		Reducer I	56,695 (7.5)		1,043	351,705	3.0	1.25 (1.17-1.34)	
		Reducer II	107,389 (14.2)		1,026	671,823	1.5	1.04 (0.97-1.11)	
		Sustainer	363,628 (48.1)		3,323	2,275,321	1.5	1 (Ref.)	
		Increaser	118,609 (15.7)		1,288	740,071	1.7	1.08 (1.01-1.15)	
Female	All Smokers (N=33,063)	Quitter	4,811 (14.6)		94	30,277	3.1	0.88 (0.70-1.11)	
		Reducer I	4,072 (12.3)		148	25,249	5.9	1.15 (0.94-1.40)	
		Reducer II	4,501 (13.6)		136	28,114	4.8	1.21 (0.99-1.48)	
		Sustainer	12,765 (38.6)		313	79,961	3.9	1 (Ref.)	
		Increaser	6,914 (20.9)		209	43,154	4.8	1.10 (0.92-1.31)	
Vascular dementia									
Male	All Smokers (N=756,469)	Quitter	110,148 (14.6)		231	692,130	0.3	0.83 (0.72-0.97)	0.797
		Reducer I	56,695 (7.5)		206	351,705	0.6	1.26 (1.08-1.48)	
		Reducer II	107,389 (14.2)		234	671,823	0.3	1.05 (0.91-1.22)	
		Sustainer	363,628 (48.1)		769	2,275,321	0.3	1 (Ref.)	
		Increaser	118,609 (15.7)		311	740,071	0.4	1.20 (1.05,1.37)	
Female	All Smokers (N=33,063)	Quitter	4,811 (14.6)		14	30,277	0.5	0.94 (0.52-1.72)	
		Reducer I	4,072 (12.3)		26	25,249	1.0	1.54 (0.95-2.49)	
		Reducer II	4,501 (13.6)		22	28,114	0.8	1.37 (0.82-2.29)	
		Sustainer	12,765 (38.6)		45	79,961	0.6	1 (Ref.)	
		Increaser	6,914 (20.9)		31	43,154	0.7	1.17 (0.74-1.85)	

© 2023 Jeong S-M et al. *JAMA Network Open.*

IR, incidence rate; aHR, adjusted hazard ratio; CI, confidence interval.
Adjusted for age, sex, income, alcohol, regular exercise, residence, hypertension, diabetes mellitus, dyslipidemia, chronic kidney disease, and body mass index.
Reducer I (≥50% reduction), reducer II (20-50% reduction), sustainer sustainers (less than 20% reduction or less than 20% increase), or increaser (≥20% increase) from baseline cigarette per day.

© 2023 Jeong S-M et al. *JAMA Network Open.*

eTable 9. Association Between Relative Changes in Cigarette Smoking Intensity and Risk of Dementia Stratified by Alcohol Drinking Status

Subgroup	Smoking status				Sustained smoking at same level as a reference				
	2009	2011	No. (%)		Case No.	Duration	IR	aHR (95% CI)	p for interaction
All Dementia (Total)									
Alcohol drinker	All Smokers (N=203,562)	Quitter	32,916 (16.2)		881	204,776	4.3	0.84 (0.77-0.90)	<0.001
		Reducer I	17,800 (8.7)		753	109,090	6.9	1.27 (1.17-1.37)	
		Reducer II	28,038 (13.8)		636	174,356	3.6	1.10 (1.02-1.18)	
		Sustainer	93,169 (45.8)		2,000	579,717	3.4	1 (Ref.)	
		Increaser	31,639 (15.5)		897	196,038	4.6	1.10 (1.03-1.18)	
Non-drinker	All Smokers (N=585,970)	Quitter	82,043 (14.0)		849	517,631	1.6	1.02 (0.95-1.11)	
		Reducer I	42,967 (7.3)		832	267,864	3.1	1.24 (1.14-1.35)	
		Reducer II	83,852 (14.3)		934	525,581	1.8	1.01 (0.92-1.10)	
		Sustainer	283,224 (48.3)		2,958	1,775,565	1.7	1 (Ref.)	
		Increaser	93,884 (16.0)		1,172	587,187	2.0	1.13 (1.05-1.22)	
Alzheimer's dementia									
Alcohol drinker	All Smokers (N=203,562)	Quitter	32,916 (16.2)		702	204,776	3.4	0.82 (0.75-0.90)	<0.001
		Reducer I	17,800 (8.7)		585	109,090	5.4	1.23 (1.12-1.35)	
		Reducer II	28,038 (13.8)		506	174,356	2.9	1.06 (0.98-1.16)	
		Sustainer	93,169 (45.8)		1,499	579,717	2.6	1 (Ref.)	
		Increaser	31,639 (15.5)		662	196,038	3.4	1.07 (0.98-1.16)	
Non-drinker	All Smokers (N=585,970)	Quitter	82,043 (14.0)		612	517,631	1.2	1.08 (0.99-1.18)	
		Reducer I	42,967 (7.3)		606	267,864	2.3	1.26 (1.15-1.39)	
		Reducer II	83,852 (14.3)		656	525,581	1.2	1.06 (0.96-1.17)	
		Sustainer	283,224 (48.3)		2,137	1,775,565	1.2	1 (Ref.)	
		Increaser	93,884 (16.0)		835	587,187	1.4	1.10 (1.00-1.21)	
Vascular dementia									
Alcohol drinker	All Smokers (N=203,562)	Quitter	32,916 (16.2)		101	204,776	0.5	0.84 (0.70-1.01)	0.147
		Reducer I	17,800 (8.7)		97	109,090	0.9	1.32 (1.09-1.60)	
		Reducer II	28,038 (13.8)		77	174,356	0.4	1.20 (1.01-1.42)	
		Sustainer	93,169 (45.8)		289	579,717	0.5	1 (Ref.)	
		Increaser	31,639 (15.5)		139	196,038	0.7	1.13 (0.96-1.33)	
Non-drinker	All Smokers (N=585,970)	Quitter	82,043 (14.0)		144	517,631	0.3	0.84 (0.67-1.05)	
		Reducer I	42,967 (7.3)		135	267,864	0.5	1.23 (0.98-1.55)	
		Reducer II	83,852 (14.3)		179	525,581	0.3	0.87 (0.68-1.12)	

© 2023 Jeong S-M et al. *JAMA Network Open.*

		Sustainer	283,224 (48.3)		525	1,775,565	0.3	1 (Ref.)	
		Increaser	93,884 (16.0)		203	587,187	0.3	1.28 (1.05-1.57)	

IR, incidence rate; aHR, adjusted hazard ratio; CI, confidence interval.
Adjusted for age, sex, income, alcohol, regular exercise, residence, hypertension, diabetes mellitus, dyslipidemia, chronic kidney disease, and body mass index.
Reducer I (≥50% reduction), reducer II (20-50% reduction), sustainer sustainers (less than 20% reduction or less than 20% increase), or increaser (≥20% increase) from baseline cigarette per day

© 2023 Jeong S-M et al. *JAMA Network Open*.

eFigure 1. Graphical Abstract of the Study

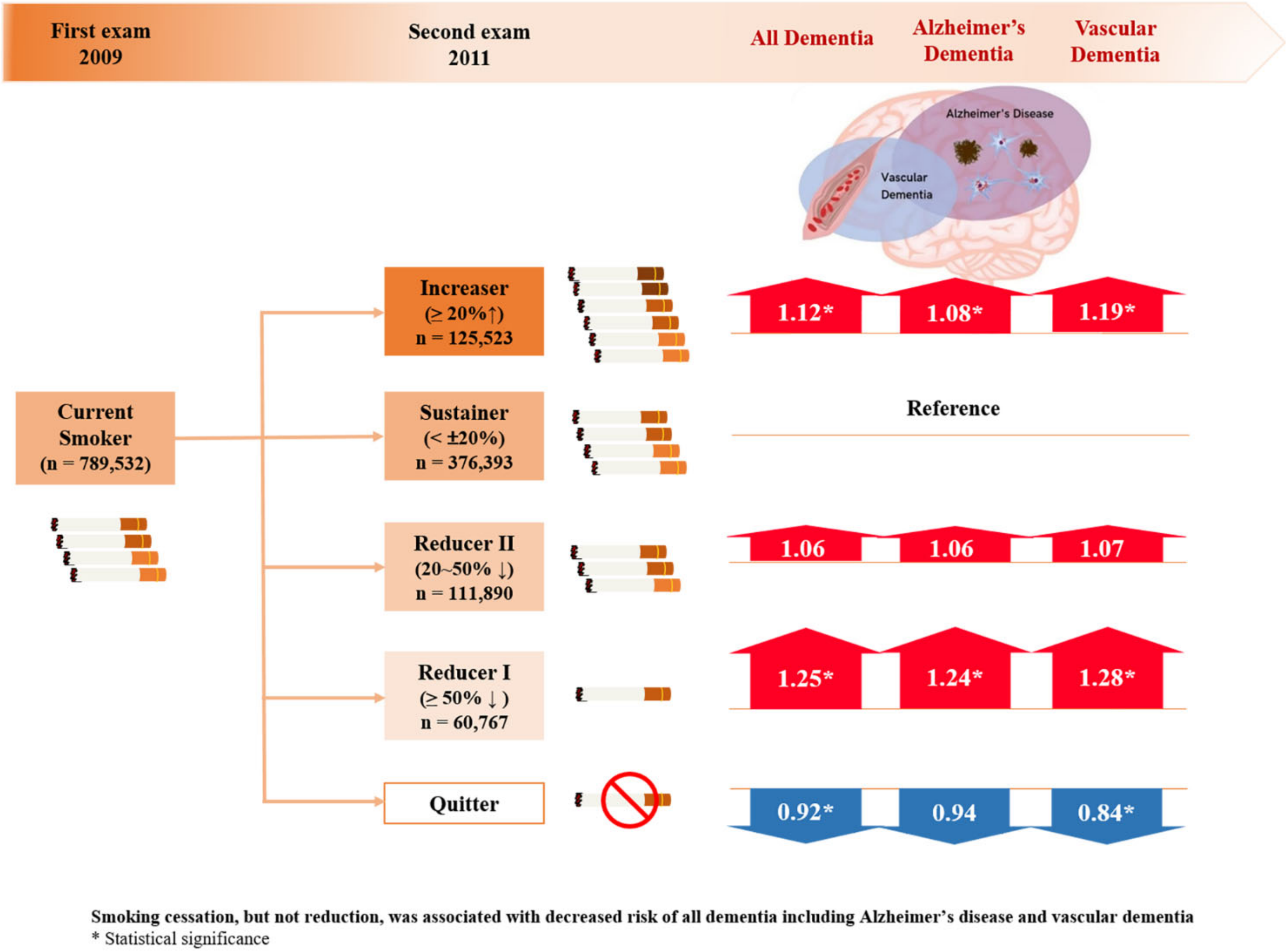

© 2023 Jeong S-M et al. *JAMA Network Open.*

eFigure 2. Restricted Cubic Spline Curve for Association Between Changes in Smoking Intensity With Risk of Dementia

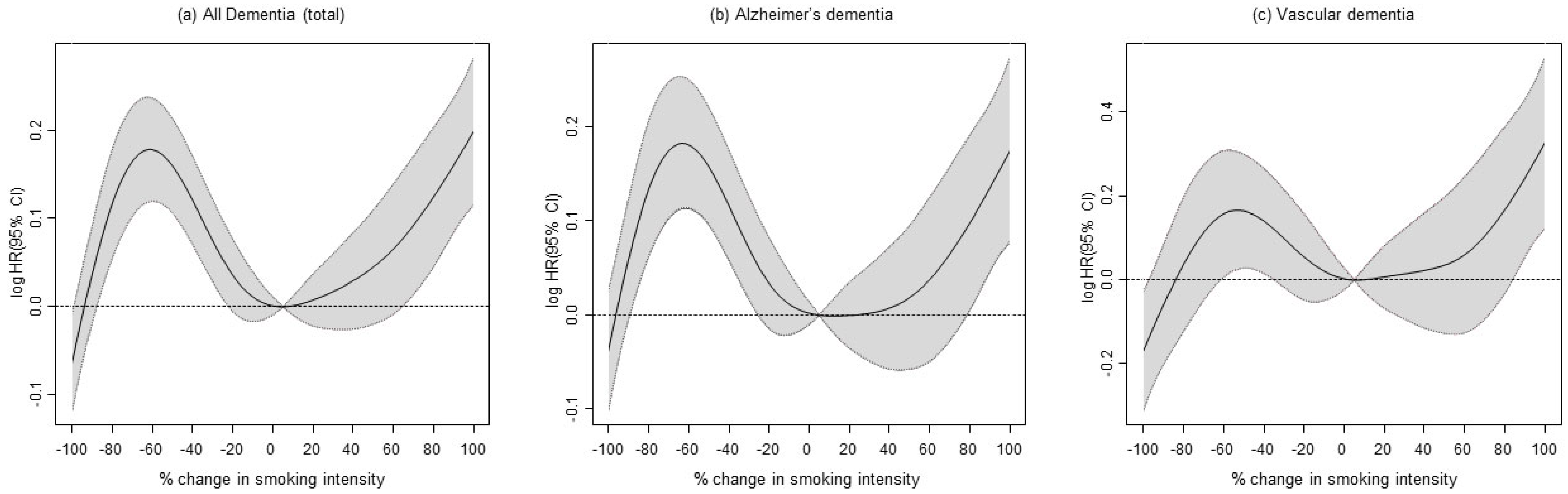

© 2023 Jeong S-M et al. *JAMA Network Open.*
