# Supplementary material for: Association of Changes in Smoking Intensity With Risk of Dementia in Korea
Source: JAMA Netw Open. 2023 Jan 19;6(1):e2251506. doi: 10.1001/jamanetworkopen.2022.51506 (PMC9857334; doi:10.1001/jamanetworkopen.2022.51506)
Supplement: Supplement 2. — Data Sharing Statement [file jamanetwopen-e2251506-s002.pdf]

Data Sharing Statement

Jeong. Association of Changes in Smoking Intensity With Risk of Dementia in Korea. *JAMA Netw Open*. Published January 19, 2023. doi:10.1001/jamanetworkopen.2022.51506

Data
Data available: No
